# Reduced Nitrogen Fertilizer Combined with Organic Fertilizer Affects Growth and Soil Physicochemical Properties of *Sapindus delavayi* (Franch.) Radlk.

**DOI:** 10.3390/plants15162509

**Published:** 2026-08-20

**Authors:** Fangyun Guo, Yi Luo, Yu Chen, Guangyu Qin, Xiaoyu Liu, Lianchun Wang

**Affiliations:** College of Forestry, Southwest Forestry University, Kunming 650224, China; fangyunguo@swfu.edu.cn (F.G.); luoyi@swfu.edu.cn (Y.L.); chenyu@swfu.edu.cn (Y.C.); qinguangyu@swfu.edu.cn (G.Q.); liuxiaoyu@swfu.edu.cn (X.L.)

**Keywords:** fertilization, growth, physicochemical properties, regulatory relationship

## Abstract

*Sapindus delavayi* (Franch.) Radlk. is a non-wood tree species of considerable ornamental, ecological, and medicinal value, and its fruits are rich in saponins, with notable cleansing and skin-care properties. However, this tree species is currently facing the dilemma of low fruit yield and unstable fruiting. Fertilization is an effective measure to improve this cultivation situation. Considering the harm of nitrogen fertilizer in soil, we designed four schemes to replace nitrogen fertilizer with organic fertilizers in this study, aiming to obtain the optimal fertilization combination for the growth of *Sapindus delavayi* (Franch.) Radlk. Three-year-old seedlings were subjected to applications of nitrogen fertilizer, organic fertilizer, and their combinations to evaluate their effects on plant physiological responses and soil physicochemical properties. Results revealed that the sole application of organic fertilizer significantly promoted the elongation of new shoots. Compared with the unfertilized control, the combined treatment of 30% organic fertilizer and 70% nitrogen fertilizer significantly increased soil nitrate nitrogen content by 161.54%, while ammonium nitrogen content decreased by 24.30%. Principal component analysis indicated that glutamine synthase (GS), glutamate synthase (GOGAT), and nitrate reductase (NR) were the major enzymes influencing leaf physiological responses. Therefore, we concluded that fertilization has affected the rate of nitrogen assimilation and altered the process of amino acid synthesis, thereby influencing the metabolism and maintenance of cellular function in *Sapindus delavayi* (Franch.) Radlk. Structural equation modeling further indicated that fertilization primarily influenced total carbon content in plant leaves by affecting soil organic matter and alkali-hydrolyzable nitrogen. These findings elucidate the regulatory relationship between plant physiological processes and soil properties under co-application of nitrogen and organic fertilizer, providing a scientific reference for field fertilization management of this tree species.

## 1. Introduction

Nitrogen is a key nutrient limiting crop growth and yield formation. When nitrogen is deficient in the soil nitrogen pool, nitrogen addition stimulates vegetative growth in crops [1]. Crops absorb and utilize nitrogen more efficiently than most other essential elements because nitrogen is directly involved in the biosynthesis of amino acid, protein, endoplasmic reticulum components, and nucleoside, and it also plays a critical role in photosynthesis and respiration [2]. These metabolic processes are not only essential for crop growth and morphogenesis; they also contribute to signal transduction and disease resistance [3]. Nitrate nitrogen and amine nitrogen are the two primary forms of nitrogen utilized for plants, with most crops preferentially assimilating nitrogen through nitrate uptake [2]. Plant roots absorb nitrate ions (NO_3_^−^) from the soil [4,5], where nitrate reductase (NR) reduces nitrate to ammonium (NH_3_). Subsequently, glutamine synthase (GS) utilizes energy (ATP) produced through photosynthesis to synthesize NH_3_ into glutamine [6]. Glutamate and glutamine are the primary metabolites involved in nitrogen assimilation, and their synthesis is jointly determined by the internal nitrogen demand and external environment al factors [1,7]. Previous studies indicate that moderate nitrogen addition promotes crop yield formation, while insufficient or excessive application leads to yield reduction or quality deterioration [8]. Therefore, the primary objective of nitrogen management is to rationally enhance soil nitrogen-use efficiency (NUE), while simultaneously maintaining crop yield and quality. However, excessive nitrogen fertilizer inputs have been reported to cause crop lodging, soil acidification, and soil compaction, therefore adversely affecting crop yields and the environment [9].

When nitrogen fertilizers are released, the application of additional organic fertilizer can significantly enhance soil physicochemical properties [10], such as the cations exchange capacity, pH, water, and aeration [11]. In addition, organic inputs increase bacterial and fungal populations by improving soil organic matter, available phosphorus, and available potassium levels [12]. As the populations of microorganism increases, organic fertilizer is fully decomposed, and its nutrients are further released [13,14]. However, the organic carbon and nitrogen in organic fertilizers are converted into inorganic forms through mineralization, and thereby the inorganic form in organic fertilizer becomes available for crops, and the slower carbon and nitrogen mineralization in organic fertilizer alone often fail to meet the nutrient demands of crops during their rapid growth period [15]. 

Microbial-mediated mineralization is a crucial process in the conversion of organic nitrogen to inorganic nitrogen (ammonium and nitrate), with mineralized nitrogen accounting for more than 20% of the nitrogen supply for annual crops [16]. Ammonification is the primary step in mineralization, whereby soil bacteria and fungi decompose protein in fertilizers into amino acids, which are then deaminated to release ammonium nitrogen [16]. Nitrification is the second step. Un well-aerated soil, ammonium nitrogen is oxidized by nitrite-oxidizing and nitrate-oxidizing bacteria into nitrate nitrogen, which is subsequently absorbed and utilized by crops [17]. Research has shown that the essence of applying organic fertilizer is to supply organic carbon (organic matter); the proportion of organic fertilizer is positively correlated with organic carbon content; and the mineralization of organic carbon is influenced by factors such as soil temperature, moisture content, pH, oxygen content, and microbial activity [15]. 

Compared with chemical fertilizers, organic fertilizers contain a large amount of organic matter, beneficial microorganisms, and a wide range of essential nutrients required by crops [7,18]. The scientific application of organic fertilizers in orchards can improve soil structure and activate soil’s microbial communities, thereby promoting the absorption of soil nutrients by the root [12]. Studies on citrus [8], wheat [19], and vegetable [9] have demonstrated that the co-application of nitrogen and organic fertilizers can increase soil nutrient levels, therefore boosting crop yields and improving quality [13]. Collectively, existing evidence indicates that the combined use of nitrogen and organic fertilizers has proven to be an effective fertilization strategy for sustainable orchard production [20].

*Sapindus delavayi* (Franch.) Radlk. is a variety of *Sapindus mukorossi* native to the Chinese provinces of Yunnan, Sichuan, Guizhou, and Hubei. This species possesses soap-making, oil-producing, and medicinal properties [21]. Currently, research on this species has primarily focused on the collection and evaluation of germplasm resources [22,23], whilst studies on fertilization in its cultivation remain relatively limited. Appropriate application of nitrogen fertilizer has been demonstrated to improve leaf photosynthetic efficiency and enhance the physiological processes of plant carbon and nitrogen metabolism [24]. Moreover, carbon and nitrogen metabolism are crucial for the growth and biosynthesis of saponins and oils in *S. delavayi* [24]. Therefore, we hypothesized that the co-application of nitrogen and organic fertilizer affects the growth of *S. delavayi*.

Based on the above knowledge, nitrogen and organic fertilizers were co-applied to *Sapindus delavayi* (Franch.) Radlk. to identify optimal fertilization strategy for this species according to growth response. First, growth parameters, such as seedling diameter, plant height, and shoot length, were measured to evaluate the effects of fertilization on plant growth. Second, soil nitrate nitrogen, ammonium nitrogen, and plant nitrogen assimilation-related enzyme activities were measured to elucidate nitrogen uptake and assimilation process following fertilization. Finally, structural equation modeling was used to analyze the cycling mechanism of soil organic matter, total carbon, and total nitrogen, and their utilization by plants under different fertilization regimes. This study systematically evaluates plant growth physiology and soil nutrient utilization responses under combined nitrogen and organic fertilization, providing a theoretical foundation for precision fertilization and sustainable production of *Sapindus delavayi* (Franch.) Radlk. 

## 2. Results

### 2.1. Growth Parameters of S. delavayi Under Co-Application of Nitrogen and Organic Fertilizer 

**Figure 1:**
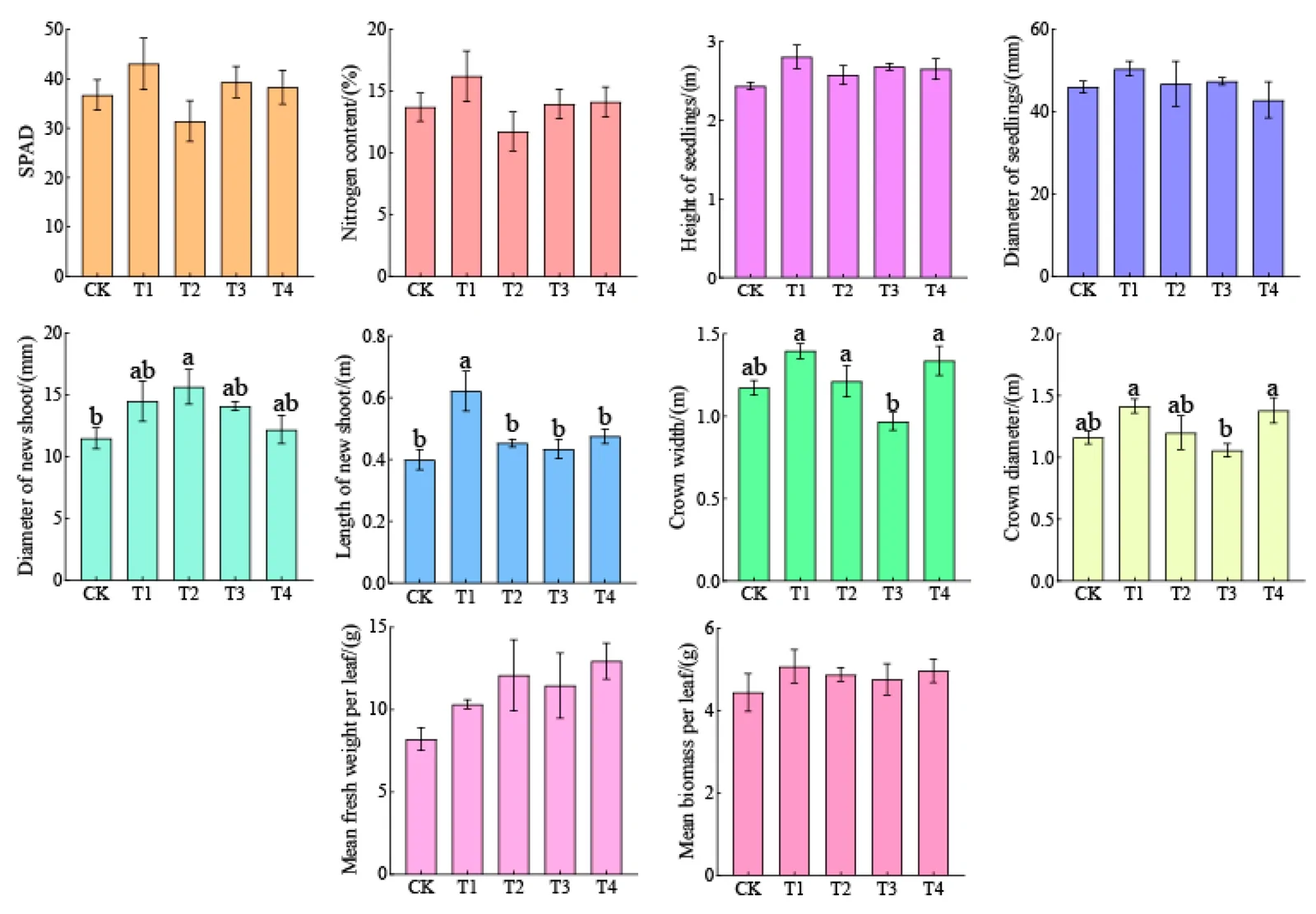
Plant growth parameters under combined application of nitrogen and organic fertilizer. Different letters on the standard errors indicate a significant difference at the 0.05 level. CK, no fertilizer; T1, 100% organic fertilizer + 0% nitrogen fertilizer; T2, 70% organic fertilizer + 30% nitrogen fertilizer; T3, 50% organic fertilizer + 50% nitrogen fertilizer; T4, 30% organic fertilizer + 70% nitrogen fertilizer.

The results showed that 6 months after fertilization, there were no significant differences among the treatments in terms of plant height, basal stem diameter, relative chlorophyll content (SPAD), and relative nitrogen content (Figure 1). However, new-shoot diameter in T2 was 36.18% higher than that in CK, and new-shoot length in T1 was 59.80% higher than that in CK. Furthermore, crown spread of T3 was 17.42% and 9.03% lower than that of CK, respectively, while that of T1 was 19.03% and 21.74% higher than CK, respectively (Figure 1). Although the fresh weight and biomass of individual leaves increased with increasing nitrogen application rates, overall, the differences among the different treatments were not significant.

**Figure 2:**
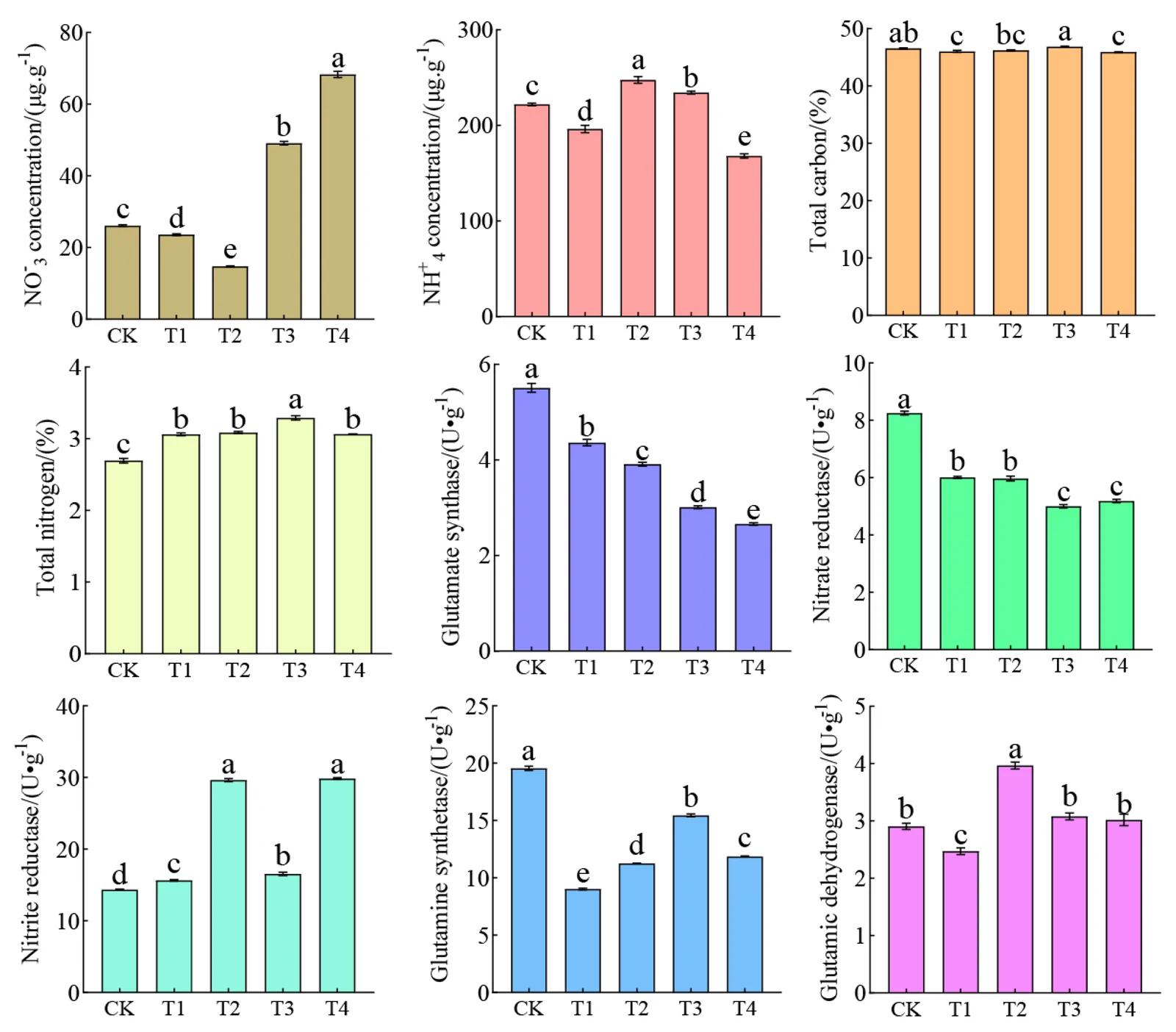
Leaf nutrient content and enzyme activity in nitrogen assimilation under co-application of nitrogen and organic fertilizer. Different letters on the standard errors indicate a significant difference at the 0.05 level. CK: No fertilizer; T1: 100% organic fertilizer + 0% nitrogen fertilizer; T2: 70% organic fertilizer + 30% nitrogen fertilizer; T3: 50% organic fertilizer + 50% nitrogen fertilizer; T4: 30% organic fertilizer + 70% nitrogen fertilizer.

Nitrate nitrogen (NO_3_^−^) content in leaves was highest in the T4 treatment, followed by T3, with levels 161.54% and 88% higher than CK, respectively; ammonium nitrogen (NH_4_^+^) content was highest in T2, followed by T3, with levels 11.52% and 5.54% higher than CK, respectively (Figure 2). Total nitrogen and carbon contents in leaves were highest in T3, with total nitrogen in T3 being 22.20% higher than in CK. Glutamate synthase (GOGAT) activities and nitrate reductase (NR) gradually decreased as the proportion of nitrogen fertilizer increased; specifically, GOGAT activity in T4 decreased by 51.68%, and NR activity in T3 decreased by 39.41% compared with CK. Nitrite reductase (NiR) activity in T2 and T4 increased by 106.54% and 108.03% compared with CK, respectively. Glutamine synthase (GS) activity in T1 decreased by 53.84% compared with CK, and glutamate dehydrogenase (GDH) in T2 increased by 36.52% compared with CK.

### 2.2. Soil Physicochemical Properties and Nutrients Under Combined Application of Nitrogen Fertilizer and Organic Fertilizer

**Figure 3:**
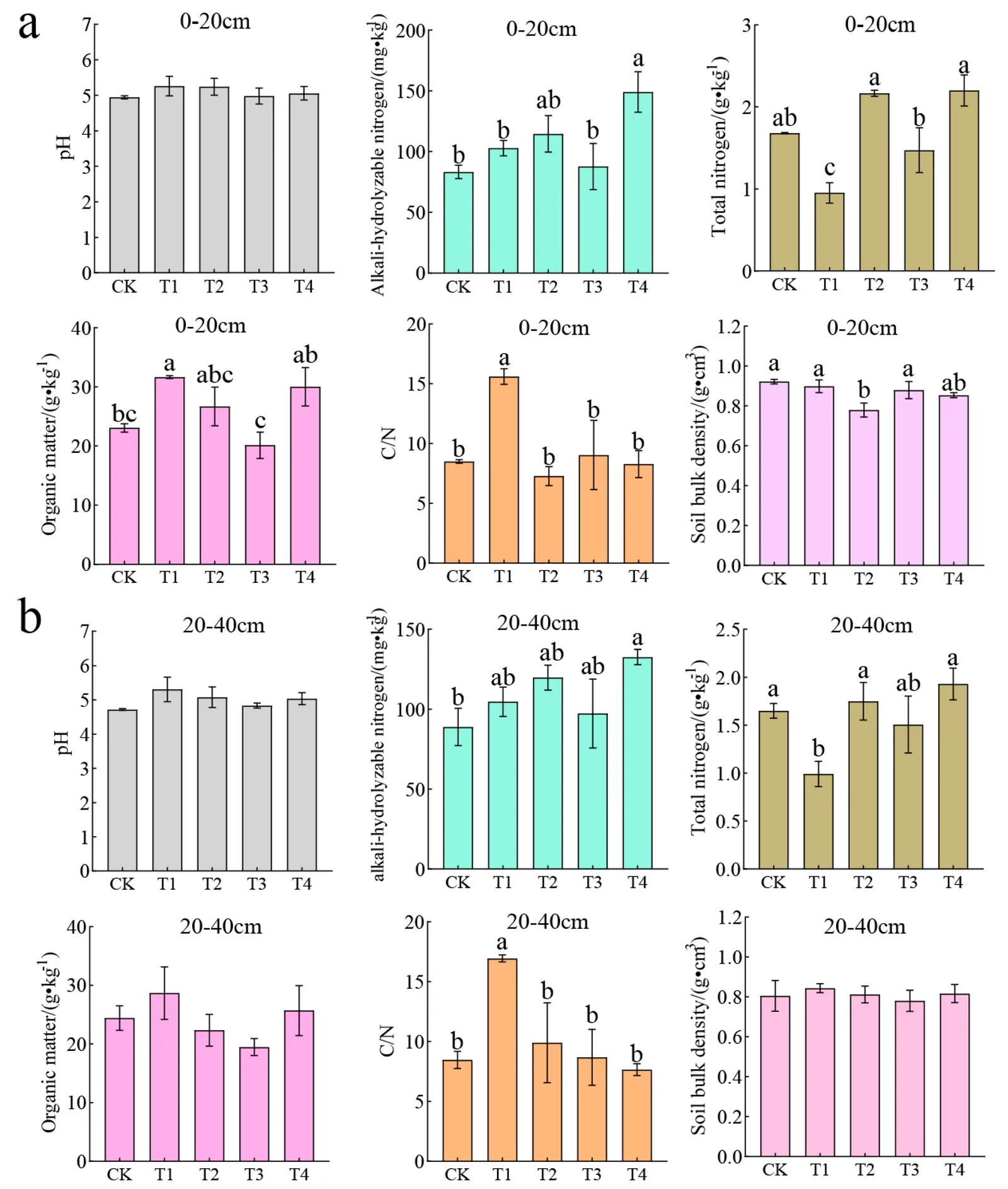
Soil physicochemical properties and nutrient content under co-application of nitrogen and organic fertilizers; (**a**) 0–20 cm soil layer; (**b**) 20–40 cm soil layer. Different letters on standard errors indicate a significant difference at the 0.05 level; the same applies below. CK, no fertilizer; T1, 100% organic fertilizer + 0% nitrogen fertilizer; T2, 70% organic fertilizer + 30% nitrogen fertilizer; T3, 50% organic fertilizer + 50% nitrogen fertilizer; T4, 30% organic fertilizer + 70% nitrogen fertilizer.

Soil physicochemical analysis indicated that pH did not show a significant response under different fertilization treatments. Alkaline-hydrolyzable nitrogen was highest in T4, where T4 increased by 49.17% compared with CK, and samples from the 0–20 cm and 20–40 cm soil layers exhibited the same trend (Figure 3). Total nitrogen content was also highest in T4 in both the 0–20 cm and 20–40 cm samples, with T4 increasing by 54.45% and 131.00% compared with CK and T1, respectively (0–20 cm). The T1 sample, which received full organic fertilizer application, had the highest organic matter content in the 0–20 cm layer, 37.23% higher than CK, while T3 (50% OF, 50% NF) had the lowest organic matter content, 12.71% lower than CK. The C/N ratio was T1 > T3 > CK > T4 > T2 in the 0–20 cm layer, while it was T1 > T2 > T3 > CK > T4 in the 20–40 cm layer; specifically, T1 was 83.56% higher than CK in the 0–20 cm and 100% higher than CK in the 20–40 cm layers. Soil bulk density in the 0–20 cm layer samples showed the order CK > T1 > T3 > T4 > T2, while in the 20–40 cm range, T1 had the highest bulk density, exceeding CK by 4.79%. It can be seen that in the 0–20 cm soil layer, the T2 treatment (30% NF + 70% OF) exhibited the highest soil porosity and the best soil texture, making it the most suitable for storing water and fertilizers.

In summary, we found that the treatment with 100% organic fertilizer (T1) significantly increased soil organic matter content and C/N ratio, while T4 treatment (70% NF + 30% OF) exhibited higher levels of alkali-hydrolyzable nitrogen and total nitrogen overall. This indicates that the soils under T1 and T4 treatments exhibited distinct differences in nutrient composition, and that soil bulk density is also influenced by the fertilization method, which may further affect the differential growth of *S. delavayi*.

### 2.3. Principal Component Analysis of Leaf Nutrient Content and Nitrogen Assimilation-Related Enzyme Activities

**Figure 4:**
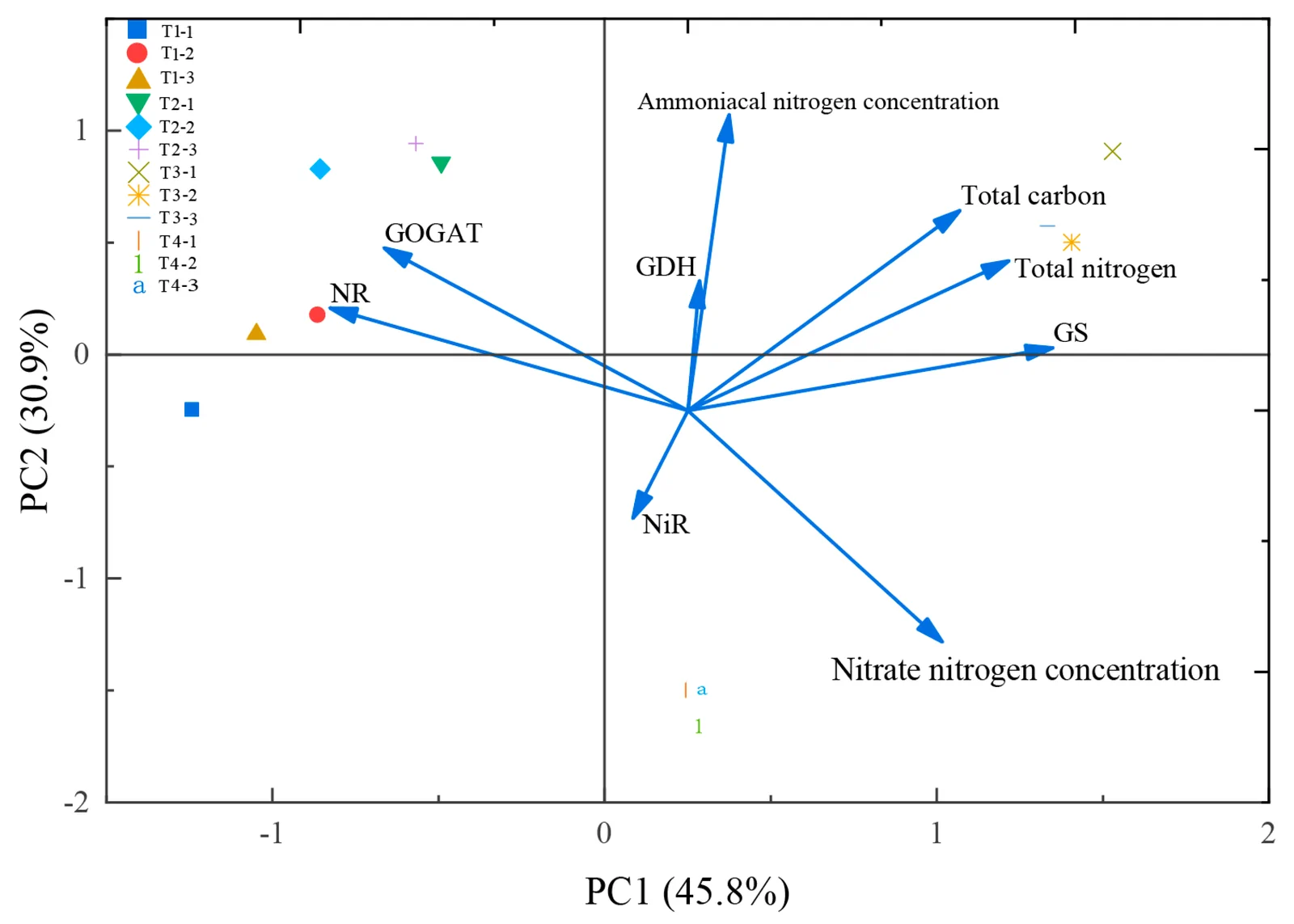
Principal component analysis of leaf nutrient content and enzyme activity. Blue arrows represent leaf nutrient parameters, and red dots represent sample sizes. GDH, glutamate dehydrogenase; GS, glutamine synthase; NR, nitrate reductase; GOGAT, glutamate synthase; NiR: nitrite reductase.

To analyze the dose-gradient response effects of co-application of nitrogen and organic fertilizers across different treatment groups, the CK with no fertilizer was removed and principal component analysis was conducted. Results indicated that glutamine synthetase (GS), glutamate synthase (GOGAT), and nitrate reductase (NR) accounted for the major physiological differences in leaves under fertilization (Figure 4). Furthermore, total carbon content in leaves was significantly negatively correlated with NiR activities, indicating that as total carbon content increased, the activities of NiR gradually decreased. 

### 2.4. Correlation Between Soil Nutrients and S. delavayi Leaf Total Nitrogen, Nitrate, and Ammoniacal Nitrogen

**Figure 5:**
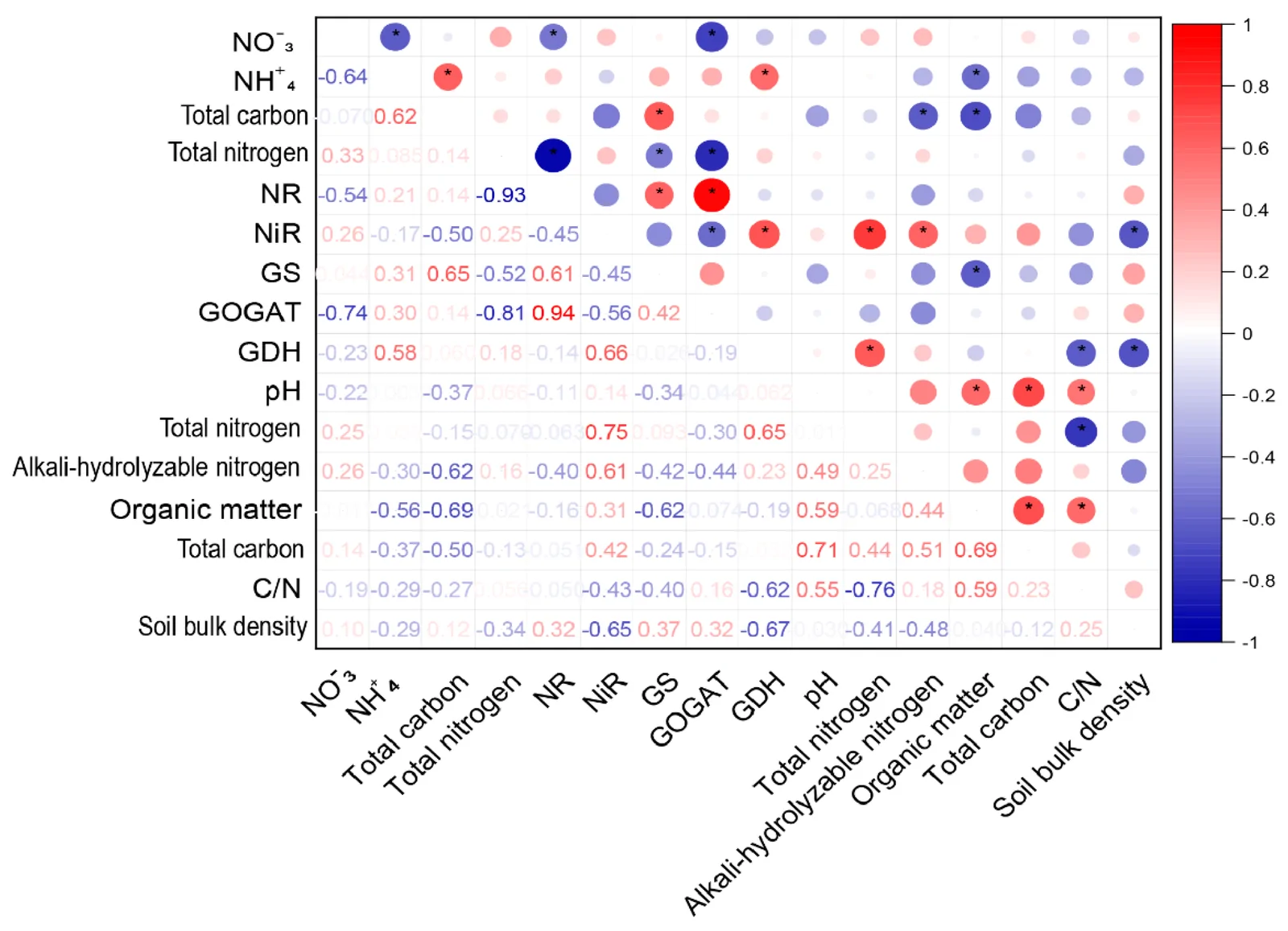
Correlation analysis between leaf and soil nutrients (red dot represents positive correlation, blue dot represents negative correlation, and * indicates a significance level of 0.05). GDH, glutamate dehydrogenase; GS, glutamine synthase; NR, nitrate reductase; GOGAT, glutamate synthase; NiR, nitrite reductase; C/N, total carbon/total nitrogen.

Correlation analysis between leaf and soil nutrient levels showed that leaves’ total carbon content was positively correlated with soil NH_4_^+^ concentration (Figure 5). Leaf glutamate dehydrogenase (GDH) showed a significant positive correlation with total soil nitrogen content, indicating that NH_4_^+^ in the soil is frequently being converted into glutamate, and that glutamate is also undergoing deamination to form α-ketoglutarate, providing a rich substrate for the formation of other amino acids. Nitrate reductase (NR) in leaves showed a significant positive correlation with glutamine synthetase (GS) and glutamate synthase (GOGAT), while nitrite reductase (NiR) showed a significant positive correlation with GDH. Total carbon content in leaves also showed a significant positive correlation with GS activity. Furthermore, total soil carbon was significantly positively correlated with soil pH and organic matter. Total soil nitrogen and alkali-hydrolyzable nitrogen were positively correlated with NiR activity. These results indicate that nitrogen addition significantly promoted the activity of nitrogen metabolism-related enzymes, reflecting that nitrogen-use efficiency was enhanced by the nitrogen-supplemented treatment.

### 2.5. Direct and Indirect Effects of Combined Application of Nitrogen and Organic Fertilizer on the Growth of S. delavayi and Soil Nutrients

**Figure 6:**
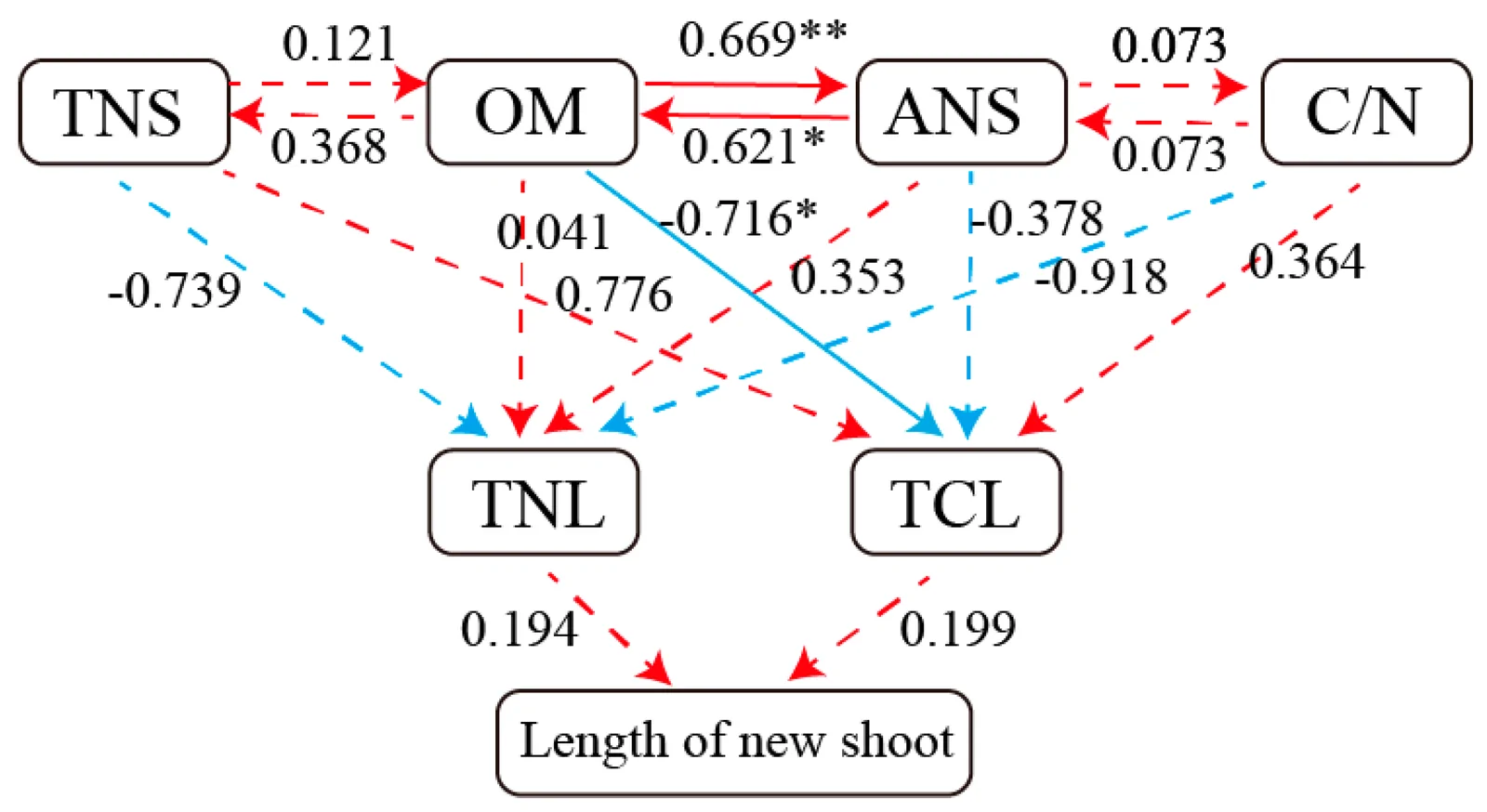
Structural equation model of the length of new shoots in *S. delavayi* under different fertilization methods (TNS, total nitrogen in soil; OM, organic matter in soil; ANS, alkali-hydrolyzable nitrogen in soil; C/N, total carbon/total nitrogen in soil; TNL, total nitrogen in leaves; TCL, total carbon in leaves). (Red arrow represents positive correlation; blue arrow represents negative correlation; dashed line represents non-significant coefficient path; number at arrow represents the path coefficient; * represents significance at the 0.05 level; and ** represents significance at the 0.01 level.).

We selected the parameters that showed the most significant responses under the fertilization treatments, namely total soil nitrogen, organic matter, alkali-hydrolyzable nitrogen, C/N ratio, and total carbon and total nitrogen in leaves, to construct direct and indirect paths through which soil conditions influence leaf and shoot growth following fertilization (Figure 6). We found that soil organic matter and alkali-hydrolyzable nitrogen levels were positively correlated, with path coefficients of 0.669 and 0.621, respectively. Furthermore, organic matter significantly suppressed total carbon content in leaves, thereby indirectly affecting plant growth. Further analysis indicated that fertilization had a smaller impact on soil organic matter than on total carbon and total nitrogen, and that organic fertilizer primarily exerted its fertilizing effect by influencing total carbon content.

## 3. Discussion

This study demonstrates that nitrogen and organic application impacted *S. delavayi* growth. Specifically, the diameter of new shoot and length of new shoot were significantly increased by 36.18% in T2 (70% nitrogen fertilizer+ 30% organic fertilizer) and 59.80% in T1 (100% organic fertilizer), respectively. This effect is primarily attributed to nitrogen’s influence on crop photosynthesis, which governs the process of carbon assimilation and further affects nutrient transport and growth [25,26]. We also found that nitrate nitrogen content in leaves was highest under T4 treatment, while ammonium nitrogen was lowest under T4 treatment, indicating that when urea is used as a nitrogen source, it promotes the conversion of nitrate nitrogen within the plant [27]. Moreover, nitrate nitrogen was negatively correlated with GOGAT and NR activity, while positively correlated with alkali-hydrolyzable nitrogen and total nitrogen in soil. This is due to the increased rate of urea hydrolysis, combined with NiR action, leading to a greater accumulation of ammonium nitrogen and total nitrogen in the soil that then becomes available for plant uptake [28]. Notably, the C/N ratio significantly rose in T1, while it was reduced in T2–T4, which indicated that net nitrogen mineralization was inhibited in T1, as in Lin’s report [15], while stimulating this process under T2–T4 treatment.

In this study, we found that there were significant correlations of varying degrees between enzyme activities involved in nitrogen metabolism, namely NR, NiR, GOGAT, and GS, and the activity of NiR and glutamate dehydrogenase was positively affected under T2 treatment (70% organic fertilizer + 30% nitrogen fertilizer), suggesting that the fertilization treatments promoted a nitrogen assimilation process through reduced nitrogen combined with organic fertilizer in *S. delavayi*. Furthermore, the activity of NR and GS in leaves gradually decreased under reduced organic fertilizer combined with increased nitrogen fertilizer, and the activities of the two enzymes were significantly correlated. This phenomenon is consistent with Pinto’s finding [29] and may be attributed to temporary metabolic changes in plant during growth phase and natural leaf ontogenesis based on plant age [30,31]. NO_3_^−^ content increased and NR activity decreased in leaves along T3–T4, and the results are in agreement with Pinto [29], which may be because nitrate accumulation in leaves occurred due to the continuous supply of NO_3_^−^ and the low capacity of NR activity to reduce this anion under T3–T4 treatment. Moreover, the inducible character of NR activity by NO_3_^−^, as mentioned earlier, seems to occur at low levels of NO_3_^−^ and is probably the reason why NR activity keeps constant under T1–T2 treatment [32].

Soil physicochemical properties indicated that fertilization increased the soil’s total carbon, total nitrogen, and organic matter content. Furthermore, positive correlations were observed between total carbon and organic matter, alkali-hydrolyzable nitrogen, total nitrogen, and pH. This may suggest that urea provides the soil with an effective nitrogen source, whilst organic fertilizer, mainly composed of sheep manure, provides abundant organic matter and organic carbon for the soil [33], thereby promoting new-shoot growth in *S. delavayi*. These findings corroborate the view that nitrogen promotes vegetative growth [34] and also indicate that the soil in our study plot is in a nitrogen-deficient state [14]. Soil reaction (pH) is an important characteristic of soils in terms of nutrient availability and plant growth [35]. The pH was positively correlated with total carbon and organic matter, alkali-hydrolyzable nitrogen, and total nitrogen content in soil, which may be attributed to the fact that carbon and nitrogen mineralization are stimulated by pH and organic matter, and soil physicochemical properties are promoted by fertilization [36].

It is well known that the combined application of organic fertilizer and various microbial inoculants can significantly promote *S. delavayi* growth [37]. The coordinated application of nitrogen, phosphorus, and potassium promoted root growth, and significantly increased the soil organic carbon and total nitrogen contents [38]. Consistent with previous research, in the present study, soil organic matter showed a significant increase in T1 (100% organic fertilizer + 0% nitrogen fertilizer); moreover, soil organic matter positively affects the alkaline-hydrolyzable nitrogen content. The organic matter in organic fertilizers can enhance soil microbial activity, ultimately improving nutrient cycling in the rhizosphere, this approach is key to enhancing soil health and plant nutrient acquisition [38]. In this study, the soil was found to be rich in organic matter and alkali-hydrolyzable nitrogen, indicating that it possessed favorable physicochemical properties and is suitable for *S. delavayi* growth after fertilizer [39,40]. Overall, fertilization influences soil organic matter and alkali-hydrolyzable nitrogen contents, thereby altering the total carbon content of plant leaves, and urea is an effective form of nitrogen for plant uptake [41]. Structural equation modeling indicates that soil organic matter directly negatively regulates the total carbon content in plant leaves, whilst positively regulating the content of soil alkali-hydrolyzable nitrogen. This may be explained by the fact that organic fertilizers containing organic matter promote the uptake and utilization of urea, a nitrogen fertilizer; however, due to the influence of the soil’s mineral ion exchange balance, the total carbon from organic fertilizer in the soil is not fully transported to the plants, resulting in a deficit in the total carbon content of the leaves [42]. Moreover, the limitation in this work is that we did not monitor the plants’ dynamic response to long-term fertilization; therefore, this is an area that warrants further investigation in the future.

## 4. Materials and Methods

### 4.1. Study Sites and Experimental Design

The experimental site was located at *Sapindus delavayi* (Franch.) Radlk. base Fuming County, Kunming City, Yunnan Province (102°21′–102°47′ E, 25°08′–25°37′ N, with an annual average temperature of 16.3 °C, frost-free period of 245 days, annual sunshine hours of 2287, annual average precipitation of 856.4 mm, evaporation of 2,032.5 mm, and relative humidity of 72%). A total of four fertilization treatments were established: T1 ((100% organic fertilizer, OF) + (0% nitrogen fertilizer, NF)), T2 (70% organic fertilizer + 30% nitrogen fertilizer), T3 (50% organic fertilizer + 50% nitrogen fertilizer), and T4 (30% organic fertilizer + 70% nitrogen fertilizer), with the untreated plot serving as the control (CK) (Table 1). In the experimental plot, row spacing was 2.5 m, and plant spacing was 3 m. Each fertilization treatment consisted of five 3-year-old *S. delavayi* seedlings, with three biological replicates per treatment, for a total of more than 80 seedlings used in the fertilization experiment. Urea was used as the nitrogen fertilizer and applied via ring-shaped trenches. The organic fertilizer, consisting of sheep manure, crop straw, and tobacco stalks, was purchased from Kunming Liangyi Environmental Protection Engineering Co., Ltd. (Kunming, China) The nitrogen application rate was determined based on previous work by An Fan [41] and Liu Juntao [43].

The soil at the study site had a pH of 4.49; a relative moisture content of 16.75%; a total nitrogen content of 1.286 g/kg; an alkali-hydrolyzable nitrogen content of 83.248 mg/kg; and an organic matter content of 23.067 g/kg; a total phosphorus content of 0.90 g/kg; a total potassium content of 5.11 g/kg; an available phosphorus content of 1.73 mg/kg; and a readily available potassium content of 561.07 mg/kg. The planting density of row pitch 3 m and row space 3 m for plants used in this study, and the average tree height is 2.63 m.

### 4.2. Properties of Organic Fertilizer

The organic fertilizer used in this study was sourced from Kunming Liangyi Environmental Protection Engineering Co., Ltd. It is a refined organic fertilizer, mainly consisting of crop straw, sheep manure, and tobacco dust. The organic matter content was 48%, total nutrient (N + P_2_O_5_ + K_2_O) content was 7.0%, total nitrogen (N) content was 3.35%, total phosphorus (P_2_O_5_) content was 1.46%, and potassium oxide (K_2_O) content was 2.18%. The moisture content was 14%, pH was 8.4, the mechanical impurity content was 0.01%, total arsenic (As) content was 6 mg/kg, total mercury (Hg) content was 0.08 mg/kg, total lead (Pb) content was 23 mg/kg, total chromium (Cr) content was 22 mg/kg, total cadmium (Cd) content was 0.63 mg/kg, and chloride ion (Cl^−^) content was 0.14%.

### 4.3. Determination of Growth Parameters and Enzyme Activity Related to Nitrogen Assimilation

A steel tape measure was used to measure tree height and new-shoot length, and a vernier caliper was used to measure the diameter of the main trunk and the base of the shoots. Crown length was measured using a steel tape measure. The relative chlorophyll content (SPAD) and nitrogen content of leaves were determined using an IN-YL03 chlorophyll meter (Shandong Laiyin Optoelectronics Technology Co., Ltd., Weifang, China, http://www.laiyinkeji.cn/about_5/, accessed on 7 April 2026)).

Three leaves were collected from each tree’s crown, for a total of 45 leaves per treatment. Leaves were taken back to the laboratory to measure their fresh weight and then dried at a constant temperature of 60 °C, until their dry weight remained unchanged, after which their biomass was determined. Ammonia nitrogen content was determined using the indophenol colorimetric method [44], while nitrate nitrogen content was determined using the salicylic acid method [44]. Total carbon and total nitrogen contents were determined using an elemental analyzer [45]. NR, GS, NiR, GOGAT, and GDH activities were measured following Zhou et al. [46] using kits (Solabio Technology Co., Ltd., Shanghai, China, https://www.solarbio.com/).

### 4.4. Soil Physicochemical Properties Analysis

After leaf harvesting in October, soil samples were collected from each fertilization treatment using a soil auger. For each treatment, five soil samples were randomly collected from a depth of 0–20 cm within each plot. These five samples were then thoroughly mixed to form a single biological sample for each treatment, with a total of three biological replicates. Soil samples from the 20–40 cm depth layer were collected using the same method described above. After returning the soil samples to the laboratory, they were dried at room temperature. A 1 mm sieve was used to separate soil from litter. Liang et al.’s [47] method was employed to determine soil alkali-hydrolyzable nitrogen, organic matter, total carbon, total nitrogen, and pH. Soil bulk density was determined following Zhu et al. [48]. Soil C/N ratio was calculated using the total carbon/total nitrogen ratio.

### 4.5. Statistical Analysis

Microsoft Excel 2010 was used to analyze the data, and SPSS20 (SPSS Inc., Chicago, IL, USA) was used to conduct one-way ANOVA and multiple comparisons (Duncan’s method). Correlation analysis and principal component analysis were conducted using Origin2021, while plant growth parameters, soil physicochemical properties, and nutrients were plotted using Graphpad Prism 8.0 (https://www.graphpad.com/). The structural equation model was conducted using the piecewiseSEM package in R 4.5.2 (IBM, Chicago, IL, USA).

## 5. Conclusions

In this work, nitrogen and organic fertilizer were applied in combination to investigate *Sapindus delavayi* (Franch.) Radlk. growth physiology and changes in soil physicochemical properties following fertilization. The results indicated that the application of organic fertilizer alone promoted the growth of new shoots, whereas the combined application of nitrogen and organic fertilizer promoted basal diameter growth. Overall, the combined application increased nitrate nitrogen content in the leaves but reduced ammonium nitrogen content. Enzyme activities related to nitrogen assimilation were primarily influenced by glutamine synthase, nitrate reductase, and glutamate synthase. Fertilization affected total carbon content in leaves by influencing soil organic matter and alkali-hydrolyzable nitrogen contents, thereby influencing *S. delavayi* growth. In brief, applying organic fertilizer alone is the most promising treatment for the growth and fruiting of the *S. delavayi*. 

## Figures and Tables

**Table 1:** Experimental design table in this study.

Treatment	Organic Fertilizer (kg/per Plant)	Urea (N:46%) (kg/per Plant)	Proportion of Organic Fertilizer (OF) and Urea (U)	Total Nitrogen Applied (kg/plant)
CK	0	0	0%OF, 0%U	0
T1	4.09	0	100%OF, 0%U	0.275
T2	2.86	0.0675	70%OF, 30%U	0.275
T3	2.05	0.1125	50%OF, 50%U	0.275
T4	1.23	0.1575	30%OF, 70%U	0.275

## Data Availability

The data referred to this article is included in the article and supplementary material.

## Supplementary Materials

The following supporting information can be downloaded at: https://www.mdpi.com/article/10.3390/plants15162509/s1.
